# New insights on the interface between metal oxide and biosystem

**DOI:** 10.1186/1758-2946-6-S1-O20

**Published:** 2014-03-11

**Authors:** Walter Langel, Susan Köppen, Wenke Friedrichs, Armin Marx, Bastian Ohler

**Affiliations:** 1 Institut für Biochemie, Universität Greifswald, 17487 Greifswald, Germany; 2 Hybrid Materials Interfaces Group, Faculty of Production Engineering University of Bremen, 28359 Bremen, Germany

## 

The interface between titanium dioxide and biological solution is relevant for the bioactivity of titanium implants. Adhesion phenomena between inorganic solids are of general interest for mineralization and bone formation processes.

Such interfaces are complex systems with many constituents including the hydroxylated metal oxide support, hydrocarbon contamination, ionic water solution and a variety of biopolymers. Our computational methods comprise classical molecular dynamics with around 10^6^ atoms in the 10-100 ns range and ab initio molecular dynamics wit 100-200 atoms for some ps.

• Electronic structure calculations show that even very thin layers of TiO^2^ on Ti metal may be commensurate and crystalline [1]. A variety of smooth and rough surfaces are implemented in force field simulations.

• The oxide is hydroxylated and its surface charge density correlates with the pH-value. The highly hydrophilic TiO^2^ is in practice screened by hydrocarbons, which may enhance inflammatory complications of implants [2]. Simulations on the nature of this contamination are presented.

• Sequence specific protein adsorption on inorganic surfaces was found in several experiments on inorganic surfaces, even though no key-lock mechanism is conceivable. We propose two effects [3]: (i) Contacts of single amino acid side chains to local charges in the surface have rupture energies, which sensitively depend on the electrostatics. The adhesion of appropriate double contacts is very strong exceeding simple hydrogen bonding. (ii) Soft motifs of proteins easier attach to the surface than rigid helices or strands.

• Close to solids water has an ordered structure, which only slightly depends on the surface charge density. These layers hinder protein adsorption. A major difference to the bulk is the reduced water mobility there.
